# Computational Modelling of Genome-Side Transcription Assembly Networks Using a Fluidics Analogy

**DOI:** 10.1371/journal.pone.0003095

**Published:** 2008-08-28

**Authors:** Yousry Y. Azmy, Anshuman Gupta, B. Franklin Pugh

**Affiliations:** 1 Department of Mechanical and Nuclear Engineering, The Pennsylvania State University, University Park, Pennsylvania, United States of America; 2 Department of Academic Services & Emerging Technologies, The Pennsylvania State University, University Park, Pennsylvania, United States of America; 3 Center for Gene Regulation, Department of Biochemistry and Molecular Biology, The Pennsylvania State University, University Park, Pennsylvania, United States of America; Max Planck Institute for Evolutionary Anthropology, Germany

## Abstract

Understanding how a myriad of transcription regulators work to modulate mRNA output at thousands of genes remains a fundamental challenge in molecular biology. Here we develop a computational tool to aid in assessing the plausibility of gene regulatory models derived from genome-wide expression profiling of cells mutant for transcription regulators. mRNA output is modelled as fluid flow in a pipe lattice, with assembly of the transcription machinery represented by the effect of valves. Transcriptional regulators are represented as external pressure heads that determine flow rate. Modelling mutations in regulatory proteins is achieved by adjusting valves' on/off settings. The topology of the lattice is designed by the experimentalist to resemble the expected interconnection between the modelled agents and their influence on mRNA expression. Users can compare multiple lattice configurations so as to find the one that minimizes the error with experimental data. This computational model provides a means to test the plausibility of transcription regulation models derived from large genomic data sets.

## Introduction

The contribution of transcriptional regulatory proteins to the expression of every gene in a genome depends upon the DNA regulatory sequences present at each gene and the physiological environment of the cell. One of the challenges in genomics, systems biology, and the study of how genes are regulated is the integration of the myriad of regulatory interactions into a meaningful network [1]. Current intuitive approaches can handle a small number of parameters, but become unwieldy as the complex interrelationships of gene regulation are expanded. Moreover, with the advent of microarray technologies that allow the RNA output of thousands of genes to be monitored in a single experiment, it becomes increasingly more difficult to interpret and integrate thousands of output values and their changes, when the system is perturbed in multiple distinct ways.

Biological networks have been thought of in at least three categories. Genetic networks describe an unfolding cascade of gene expression events in which one or more genes influence the expression of other genes that go on to influence the expression of more genes [2], [3]. Protein networks describe the set of all measurable protein-protein interactions within a cell. Biochemical networks describe the flow of metabolites from one enzyme to another [4], [5], [6]. Our goal here is to integrate parts of these networks with respect to the biochemical assembly of the transcription machinery at eukaryotic promoters, resulting in the conversion of nucleotide substrates into RNA.

Genes represent the source code for the components of a cell. When individual genes are “read”, nucleotide substrates are converted to an RNA polymer product. Using a fluidics analogy, one can think of a pipe lattice in which a single fluid, mRNA, flows at rates determined by properties of the pipes in the lattice and subject to the influence of individual valves placed, by the modeller, on selected pipes in the lattice. The single fluid enters/exits the lattice from external nodes driven by a pressure head whose value is part of the model specification. A valve on a pipe controls the flow through that pipe, and in the fluidics analogy the net effect of all valves in the lattice controls RNA output. Biologically, the valves correspond to proteins that control the assembly/disassembly of the transcription machinery at the beginning of genes (promoters). Once assembled, the RNA polymerase II component of the transcription machinery transcribes the gene, in effect converting nucleotides to RNA. Assembly of the transcription machinery involves a wide variety of proteins, including both positive and negative regulators. Thus, a piping analogy would include many valves. Our goal is to define a pipe network analogy and its associated valves that properly model RNA output at every modelled gene.

Flux simulators, which model the movement of substrates through a reaction pathway using deterministic and/or stochastic approaches [5], [6], [7], require explicit declarations of reaction steps, rate constants, and reactant concentrations. As a result they may be less suitable for modelling the assembly of the transcription machinery, where such parameters are ill-defined. Rather, a cruder approach may be needed to model less-defined systems with the purpose of evaluating the plausibility of potential regulatory mechanisms.

**Figure 1:**
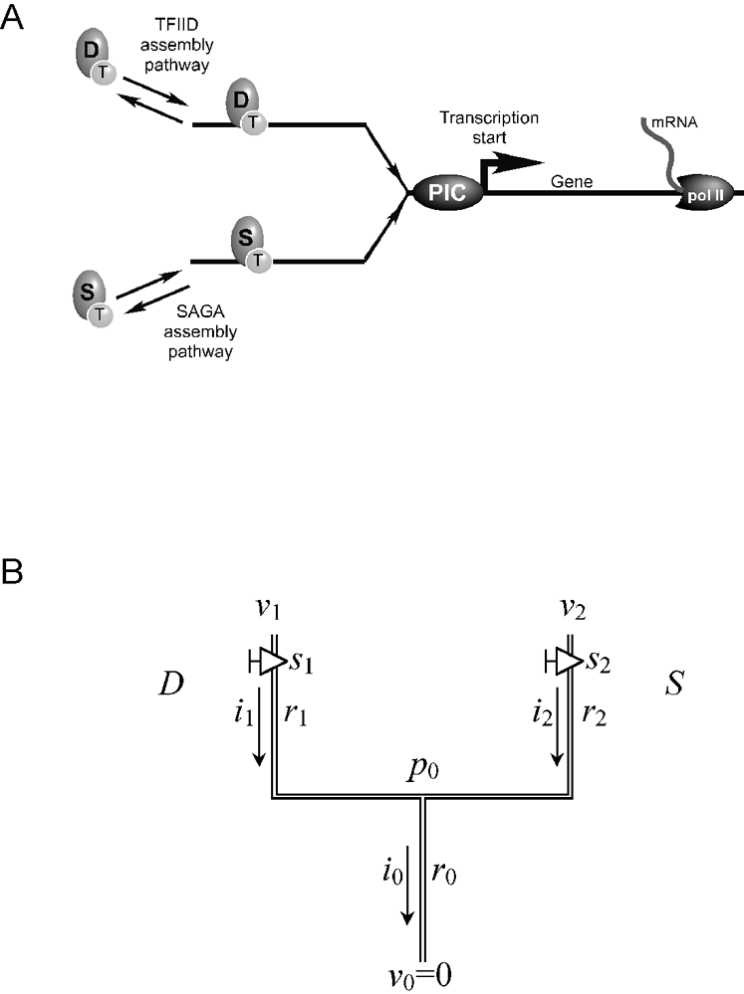
Pathways of transcription complex assembly. A, Simplified model of protein complex assembly on DNA. B, Two-branch model in which TFIID (D) and SAGA (S) compete to load TBP (T) onto DNA, which then goes on to form a pre-initiation complex (PIC).

Based upon a wide variety of biochemical, genetic, and genomic experiments and conventional wisdom, we and others (see Ref. [8], and references therein) devised a minimal framework for the assembly of the transcription machinery through the TATA binding protein (TBP) at all measurable promoters, using the budding yeast *Saccharomyces cerevisiae* as a model system. Because the transcription machinery is fundamentally conserved in the eukaryotic lineage, this framework is potentially applicable to higher eukaryotes including humans. In this framework, two protein complexes, TFIID (transcription factor IID) and SAGA (Spt Ada Gcn5 Acetyltransferase), compete to assemble the transcription machinery via recruitment of TBP to promoters (Figure 1). Consequently, they are functionally redundant, but only partially redundant since each pathway potentially produces quantitatively distinct outputs (i.e. different mRNA levels). Therefore within this framework, TFIID and SAGA provide two potential levels of control, one in which TFIID and SAGA compete for promoter binding and a second where promoter-bound TFIID or SAGA drives a quantitatively distinct mRNA output. The combined action of the physiological milieu (protein network) and promoter DNA regulatory elements further tweaks these pathways to achieve gene-specific transcriptional control.

Previously, we utilized a set of mutations that eliminated or reduced the individual contributions of TFIID and SAGA (as well as other regulators) to monitor changes in gene expression on a genome-wide scale in yeast [8], [9]. In one of those studies, we measured the effect of over 60 mutant combinations on ∼6000 yeast genes. After clustering the genes into six groups based upon similar responses to a set of mutations (i.e., co-regulation), there were 360 (60×6) summarized data points, all of which needed to be reconciled in the context of a generalized gene regulation model. Model plausibility was evaluated using a kinetic simulator [7], [10] that allowed us to define the transcription process in terms of elementary steps that were relevant to the mutants under study. While this strategy was effective in shaping an all-inclusive model, it was primarily designed to model a forward pathway in which input parameters were declared and the algorithm calculated the output (mRNA production). The process was used interactively to test whether a series of reaction steps and input parameters defining assembly of the transcription machinery could accommodate the bulk of the data. This process, while effective, was laborious because it lacked an iterative optimization routine. For this reason we sought to develop a computational optimization process by which measured mRNA output levels could be used to derive input parameters that would model the output in a model-specific manner.

TFIID and SAGA are but two of the many protein complexes that control mRNA output (Figure 1A) [9], [11], [12], [13], [14]. The ultimate goal is to create a linked biochemical network that integrates all regulatory interactions. The magnitude of such a problem is substantial if one considers that in yeast alone there are ∼6000 genes potentially regulated by up to ∼400 proteins, thereby producing ∼6000×2^400^ possible bound/unbound states. Exhaustive experimental testing of such a theoretical network would require the impractical construction of 2^400^ mutant strains to produce each state. As a tractable, albeit limited, means of elucidating parts of the network, we have focused on key components (i.e., TFIID, SAGA, and several other proteins) with the goal of creating a mathematical “fluidics” model that describes the contribution of TFIID, SAGA, and other selected proteins to genome-wide gene expression. The mathematical model is intended to evaluate the plausibility of *ad hoc* conceptual models of transcription regulation that explain changes in gene expression in response to defects in the regulatory interactions under study.

### The Fluidics Analogy Model

A biochemical network describing the assembly of the transcription machinery at promoters can be thought of as a series of reversible fluid-flows that dynamically move forward (transcription machinery assembly) and backward (disassembly or inhibition), with mRNA output being the net flux of these forward and reverse flows. To model regulated mRNA production from a gene, we developed a piping analogy (Figure 1B) in which a single fluid, namely mRNA, flows through the pipe at a rate governed by the pipe's resistance and the pressure drop across the ends of the pipe. In addition, a valve is deployed (on selected pipes in the lattice) to represent a regulatory event such as TFIID binding to a promoter. Since TFIID contributes to mRNA production [15], the valve is considered “on” when TFIID is bound at the promoter. When TFIID is experimentally removed by creating a mutation in TFIID, the valve is then “off” and mRNA output is decreased. Addition of SAGA to the system creates two inputs, or pipes, that converge to produce a single mRNA output. In this work a set of such pipes is referred to as a *lattice*, and is constructed by the user to conceptualize experimental observations. In principle, the 6000 yeast genes can be modelled by 6000 individual lattices. However, rather than creating a computationally unwieldy set of 6000 lattices, clusters of genes that behave similarly within experimental error, when “valves” are turned on/off via mutation, are approximated using a single lattice. In this paper, we describe the modelling of six clusters with six lattices. An important aspect of this model is that a valve is experimentally turned on by mutating a negative regulator or turned off by mutating a positive regulator.

Under the normal physiological state of the cell (i.e. wild type), valves will have default settings ranging from zero (off) to some maximal value (fully on). A valve in one lattice, representing a given gene cluster, may have a different default (wild type) setting than the same valve in a different lattice (representing a different gene cluster). For example, lowly expressed genes might have default valve settings for TFIID close to zero. Highly transcribed genes might have the TFIID valve set to a high value. When TFIID is mutated so as to turn off all TFIID valves, mRNA output from the lowly expressed gene is relatively unaffected, whereas mRNA output from highly transcribed genes might be severely curtailed. In a two-pipe lattice involving TFIID and SAGA, the change in mRNA output upon mutation of TFIID (or SAGA) will depend upon the default valve settings for TFIID and SAGA. Since complete elimination of certain regulatory proteins such as TFIID is lethal to cells [14], we must either measure RNA output soon after TFIID inactivation, or use TFIID mutants that are only partially defective, thereby requiring the model to tolerate a relatively high background level of RNA output when the TFIID valve is turned “off”. The first option is employed in modelling the 2-branch lattice below, and the latter when modelling the 4-branch lattice; the 2- and 4-branch lattices are described in the remainder of this paper. In either case, the effect of mutations, i.e. changing on/off valves' setting in our model, is measured as the difference of the resulting mRNA outflow from the all-valves-off state.

The resulting flow across a given valve has three possible states: no-flow, flow in the forward direction, or flow in the reverse direction depending (within the scope of the fluid-flow analogy of this model) on the pressure drop across the pipe holding the valve in question. The effect of the collective settings of all valves in a given lattice on the resulting net-outflow (i.e., mRNA production) has three possible states: increase, decrease, or no-change. Experimentally, this corresponds to a positive, negative, or no change, respectively, in mRNA levels for each gene (or gene cluster) being measured. Only the on/off states of each valve are controlled, and the corresponding net outflow is measured thereby enabling a quantitative inference of the change in flow through all valves.

Our previous study on the TFIID/SAGA assembly pathway included a third non-productive transcription complex assembly pathway [8]. This non-productive pathway loads TBP onto promoters in a state that fails to direct proper assembly of the transcription machinery. Promoter activation therefore requires removal of this inactive TBP [16], [17], which is catalyzed by the potentially cooperative action of Mot1 (Modulator of transcription 1) and NC2 (Negative cofactor 2) [16], [18], [19], [20], [21]. In this model, Mot1 and NC2 also remove TBP delivered by SAGA [8], [9], but not TBP delivered by TFIID [22]. The six clusters of co-regulated genes derived from that study are the subject of four-branched lattice modelling presented here.

### A Two-branch Pipe Network

As a first step towards modelling complex lattices, we created a simplified two-branch model representing the dual contributions of TFIID and SAGA to mRNA production (Figure 1B) [9]. This model is defined by 1) its connection scheme; 2) the pressure heads at all external nodes, *v*_0_, *v*_1_, *v*_2_; 3) the resistance to flow along each pipe link, *r*_0_, *r*_1_, *r*_2_; and 4) the on/off setting of valves *s*_1_, *s*_2_. Since the flow depends only on pressure drops between the input and output pressure heads, all pressure heads in the model can be specified relative to the output pressure head (*v*_0_) which, without any loss of generality, is set to zero for convenience. The external pressure heads and pipe resistances are fixed “model parameters” which are specific to each lattice. The measurable “output” of an experiment is the single flow variable *i*_0_ that is analogous to the production rate of mRNA for the given *s*_1_, *s*_2_ setting minus the production rate of mRNA when both valves are closed; the latter is termed the “background state”. The internal pressure head (*p*_0_), and all flow variables *i*_0_, *i*_1_, *i*_2_, are computed from the model parameters consistent with a specific setting of the valves, and in accordance with the standard fluidics model equations:
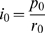
(1)

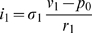
(2)

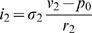
(3)where the constant *σ_i_* is assigned a value of 1 if valve *s_i_* is open (i.e. *on*), and a value 0 indicating that valve *s_i_* is in its *off* position. In addition, flow continuity at pipe connections requires:
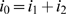
(4)The model parameter *r*_0_ defines a class of solutions under the transformation:
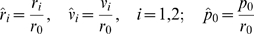
(5)Without any loss of generality, we arbitrarily set *r*_0_ = 1, recognizing that a different setting of this model parameter will scale *p*_0_ and the remaining model parameters according to Eq. (5) leaving all other model variables, most importantly *i*_0_, invariant. Simultaneously solving equations (1)–(4), with *r*_0_ set to 1, yields the expression for the output flow *i*_0_

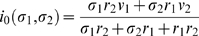
(6)The arguments (*σ*_1_, *σ*_2_) of *i*_0_ in Eq. (6) are intended to affirm the dependence of the output flow on the valves setting. It is evident from Eq. (6) that when both valves are closed *i*_0_ vanishes providing the background case against which other valve-settings' measured outflow is compared. Hence in Eqs. (1)–(6) *i*_0_(*σ*_1_, *σ*_2_) denotes the difference of the mRNA outflow for the valve setting (*σ*_1_, *σ*_2_) from the mRNA outflow when both valves are closed, i.e. *σ*_1_ = 0 = *σ*_2_.

### Optimal Solution of the Two-Branch Model for the Model Parameters

With two valves in the two-branch model, each permitting two states, *on* or *off*, there is a total of four possible valve-setting combinations available, each yielding a model value of *i*_0_ that correlates with a correspondingly measured value of mRNA relative to the background. Labeling branches 1 and 2 in Figure 1B as *D* and *S*, respectively, we designate the *wild type state* (i.e. *unperturbed* or *starting state*) as the experiment where the two valves are set to the *on* position, and set the value of *i*_0_ to the differential measured mRNA flow:
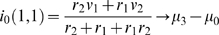
(7)(Note that, for example, the subscript 3 on *μ*_3_ is the integer represented by the binary number 11 corresponding to the valves setting for the corresponding state). In Eq. (7), *μ*_0_ is the measured value for the background type state defined above. All other three states corresponding to the remaining settings of the valves are experimentally *altered states* corresponding to differential measured mRNA flows *μ_i_*, *i* = 0,1,2:
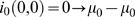
(8)

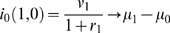
(9)

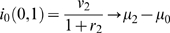
(10)
Table 1 lists these four experiments and their experimentally measured mRNA outflow for 6 clusters of yeast genes. For each cluster, within the scope of the two-branch model, there are four measured values *μ_i_*, *i* = 0,…,3, that can be substituted into the right hand sides of Eqs. (7)–(10) and the resulting relations used to determine the four model parameters: *v*_1_, *v*_2_, *r*_1_, *r*_2_. By design Eq. (8) is automatically satisfied, hence it cannot be used in the process of evaluating the model parameters. Consequently the system of equations (7), (9), and (10) is underdetermined in its unknowns, the four model parameters in this case. Thus values of the model parameters are sought that best fit the model-computed values of *i*_0_ to their measured values. This defines an optimization procedure and the optimal state was achieved by searching for the set of model parameters that minimizes the following quantities:
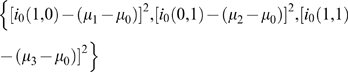
(11)under the constraints: 1) *i*_0_≥0; 2) *r_i_*≥0; and 3) the sense of change from the wild state is preserved, e.g. [*i*_0_(0,1)–*i*_0_(1,1)][*μ*_2_–*μ*_3_]>0, with an analogous constraint on Experiment 1. Within the analogy to pipe lattices it is physically acceptable to have negative pressure heads, *v_i_*, and flows, *i*_1_ and *i*_2_, but not resistances, *r_i_*. A negative resistance would imply flow from low to high pressure, an unsustainable proposition in view of the intended analogy.

**Table 1:** Four experiments available in the Two-Branch Model: Model expressions and experimentally measured values for 6 clusters.

Model *i*_0_+*μ*_0_	*SD* off	*S* off	*D* off	Wild: All on
	*μ*_0_	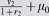	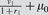	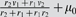
Cluster 3	0.89	2.97	1.99	2.90
Cluster 4	0.19	0.54	0.42	0.80
Cluster 5	0.54	1.87	1.68	2.30
Cluster 7	0.51	1.95	1.80	2.60
Cluster 8	0.77	2.85	2.07	3.75
Cluster 9	1.69	6.89	4.91	8.10

The optimization problem is formulated as a constrained nonlinear programming (NLP) problem where the objective is the minimization of the squared difference between the experimental and predicted differential outflow (from the background state's outflow) for all the included experiments. The resulting model is implemented using the General Algebraic Modelling System (GAMS) [23] which is a high-level language for the compact representation of mathematical programming models. Subsequently, the model is solved using the CONOPT3 solver which implements the Generalized Reduced Gradient (GRG) algorithm [24], [25]. Search procedures perform the minimization locally so as to reduce computational demand. Hence, they do not guarantee a global minimum.

Applying the GAMS [23] optimization procedure to the measured mRNA output values presented in Table 1 yields the model parameters shown in Table 2. These values correspond to the constrained minimum error stated in equation (11) obtained in 1,000 iterations designed to explore a wider region in model-parameter space thus improving the chance of approaching the global minimum at a reasonable computational cost. Each iteration is comprised of a complete minimization sequence, with the various iterations differing from one another by the values assigned as initial guess to the model parameters and variables. For example the optimal model parameters presented in Table 2 were obtained by randomly selecting an initial guess as follows:
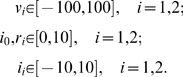
(12)These model parameters are physically acceptable in that no negative resistances appear. These optimal model parameters reproduced, within experimental error, the measured experimental output of mRNA [9] for each of the modeled six clusters [8] under each permutation of the wild type and mutant (TFIID and SAGA mutants) states (Table 3). The C∶E error is defined as the ratio of the computed value of *i*_0_ for a given valve setting to its measured value corresponding to wild/altered-type yeast minus one. The error is the maximum of |C∶E–1| over all valve settings for a given cluster. Table 3 shows that the error for all clusters is below 8.1%, well within the experimental variability. This modeling involves experimental data generated 45 min. after complete inactivation of TFIID via a temperature-shift in the mutant strain *taf*1-2.

**Table 2:** Two-Branch Model parameters.

	*v*_1_	*v*_2_	*r*_1_	*r*_2_
Cluster 3	1.93	17.13	0.74	7.12
Cluster 4	735.77	758.61	3480.6	2139.60
Cluster 5	2.48	7.43	1.28	4.72
Cluster 6	3.61	18.71	2.02	12.44
Cluster 8	21.01	89.96	17.48	43.44
Cluster 9	10.83	43.39	2.84	7.62

Parameters were obtained by minimizing the residual of equations (7), (9), and (10) using the 6 clusters experimentally measured values shown in Table 1.

**Table 3:** Modeling a two-branch pipe lattice.

Experiment	0	1	2	3	
*s*_1_ **(S)**	off	off	on	on	
*s*_2_ **(D)**	off	on	off	on	Error
Mutant	*SD*	*S*	*D*	*(WT)*	
**Cluster 3**					
Measured *i_0_*	0.89	1.99	2.97	2.90	
Calculated *i_0_*	0.89	2.00	3.00	2.90	0.011
C∶E–1	0.000	0.004	0.011	0.000	
**Cluster 4**					
Measured *i_0_*	0.19	0.42	0.54	0.80	
Calculated *i_0_*	0.19	0.40	0.54	0.76	0.056
C∶E–1	0.000	−0.040	0.001	−0.056	
**Cluster 5**					
Measured *i_0_*	0.54	1.68	1.87	2.30	
Calculated *i_0_*	0.54	1.63	1.84	2.30	0.035
C∶E–1	0.000	−0.034	−0.020	0.000	
**Cluster 6**					
Measured *i_0_*	0.51	1.80	1.95	2.60	
Calculated *i_0_*	0.51	1.71	1.90	2.60	0.051
C∶E–1	0.000	−0.051	−0.022	0.000	
**Cluster 8**					
Measured *i_0_*	0.77	2.07	2.85	3.75	
Calculated *i_0_*	0.77	1.91	2.79	3.80	0.079
C∶E–1	0.000	−0.078	−0.020	0.013	
**Cluster 9**					
Measured *i_0_*	1.69	4.91	6.89	8.10	
Calculated *i_0_*	1.69	4.51	6.73	8.10	0.081
C∶E–1	0.006	−0.081	−0.024	0.000	

* See Figure 1B for lattice arrangement. Valve settings are denoted by *s*. Mutant status is indicated by *S* (*spt*3Δ) and *D* (*taf1-2*) [9]. Error is defined as the maximum absolute value of the error obtained between the measured [9] and calculated *i*_0_ values. Measured *i*_0_ for WT (experiment 3) corresponds to the average transcription frequency (mRNA/hr) using the data of Holstege [26] for the indicated clusters of genes (3, 4, 5, 6, 8, and 9) defined in Huisinga et al. [8]. Measured *i*_0_ for experiments 0–2 is the result of the following calculation: Transcription frequencies from experiment 3 (WT), for individual clusters, were multiplied by the fold changes in transcription (linear scale) measured previously with mutants *spt*3Δ and *taf1-2* by Huisinga et al. [9]. “C∶E–1” error is defined in the text.

Effectively this optimization procedure amounts to solving the inverse problem, whereby the measured mRNA output is known and attempts are made to infer the model parameters that most closely reproduce that output. Importantly, perturbations to the experimental system via mutation are used to alter the corresponding valve setting(s). While the under-determined nature of the two-branch model is unlikely to repeat in more complex models with more branches, the optimization procedure applied to this two-branch model is equally applicable to over-determined systems. This is further illustrated for the four-branch model below.

The construction of a mathematical model governing transcription complex assembly amounts to determining all model parameters that when deployed in the pipe-lattice model will approximately produce the correct outflow *i*_0_ for all possible valve patterns (mutant states) in a given cluster. Several considerations place limits on the accuracy of this approach: 1) Inherent variance of gene expression within a cluster, 2) experimental error associated with the measured results, 3) uncertainty of the appropriate lattice connections, and 4) a “ripple” effect, whereby the effect of the primary perturbation (mutation) to the experimental system creates other perturbations that affect net mRNA output.

### A Four-Branch Model

**Figure 2:**
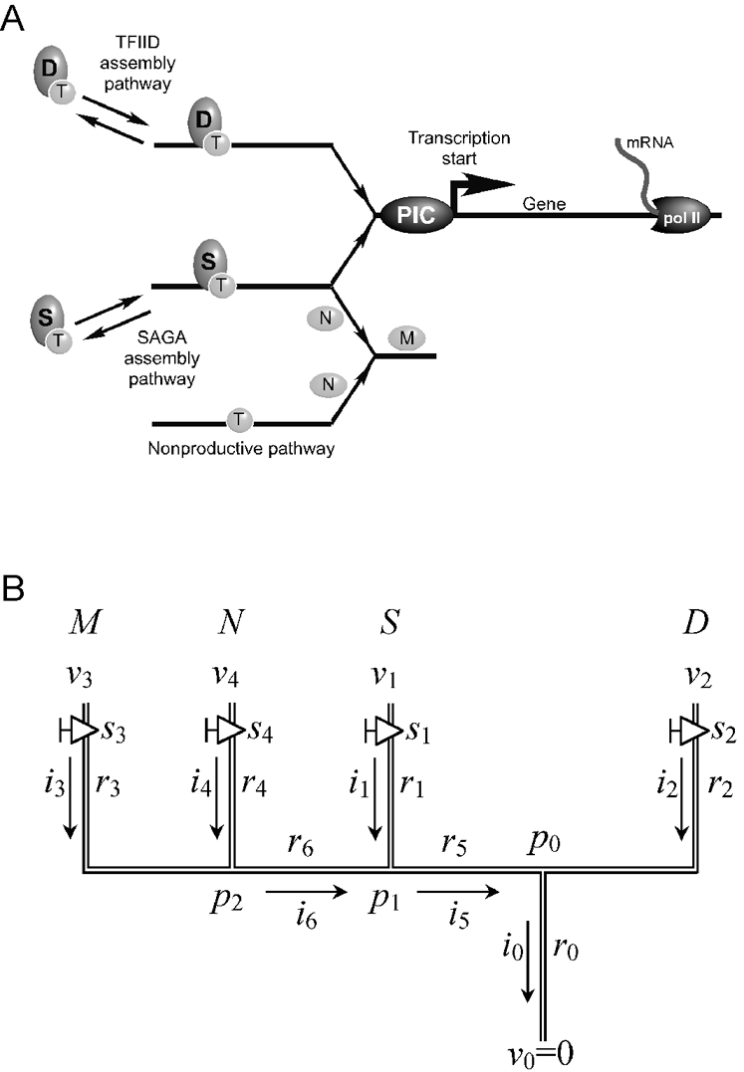
A four-branch model. A, In this pathway two inhibitory proteins NC2 (N) and Mot1 (M) inhibit the SAGA pathway and inhibit a non-productive TBP assembly pathway. In the latter case NC2 and Mot1 would act positively. B, Simple pipe lattice for illustration of the computational model parameters and variables.

Next we constructed a more complex multi-branch lattice reflecting contributions of the transcriptional regulators Mot1 and NC2 to the two-branch model involving TFIID and SAGA (Figure 2). The four-branch model's construction is based upon evidence of these interactions presented elsewhere [9]. The number of control valves was set to the number of individual mutations being modelled. Six identical lattices were constructed, each modelling one of the six previously defined clusters of co-regulated genes [9]. Each lattice was tailored by allowing its model parameters, namely the external pressure head (*v*) at each external node and the resistance of each pipe link, to vary from one cluster to the other. In addition, the model variables, flow currents (*i*) and internal pressure heads at pipe intersections (*p*) vary across clusters and with varying valves settings.

The data set used for the four-branch model is different from that used for the two-branch model. In particular, TFIID was only partially inactivated using the *taf*1(Δ*TAND*) mutation, and all mutant states were constitutive (i.e. not induced by a temperature shift, as in the previous example). Consequently, in the all-off background state, a relatively high level of background mRNA remains.

The four-branch model is “over-determined” in that there are more conditions to satisfy (measured output from experiment) than model parameters to compute. This feature derives from the fact that the number of model parameters (i.e. number of pipes and external nodes) increases linearly with lattice complexity, while the number of valve-setting combinations increases exponentially, i.e. 2*^K^*, with the number of valves, *K*. An optimal set of model parameters is sought that minimizes the deviation of the computed values of *i*_0_ under various valve settings from their corresponding experimentally measured values. Additional constraints placed on the optimization procedure are described below.

**Figure 3:**
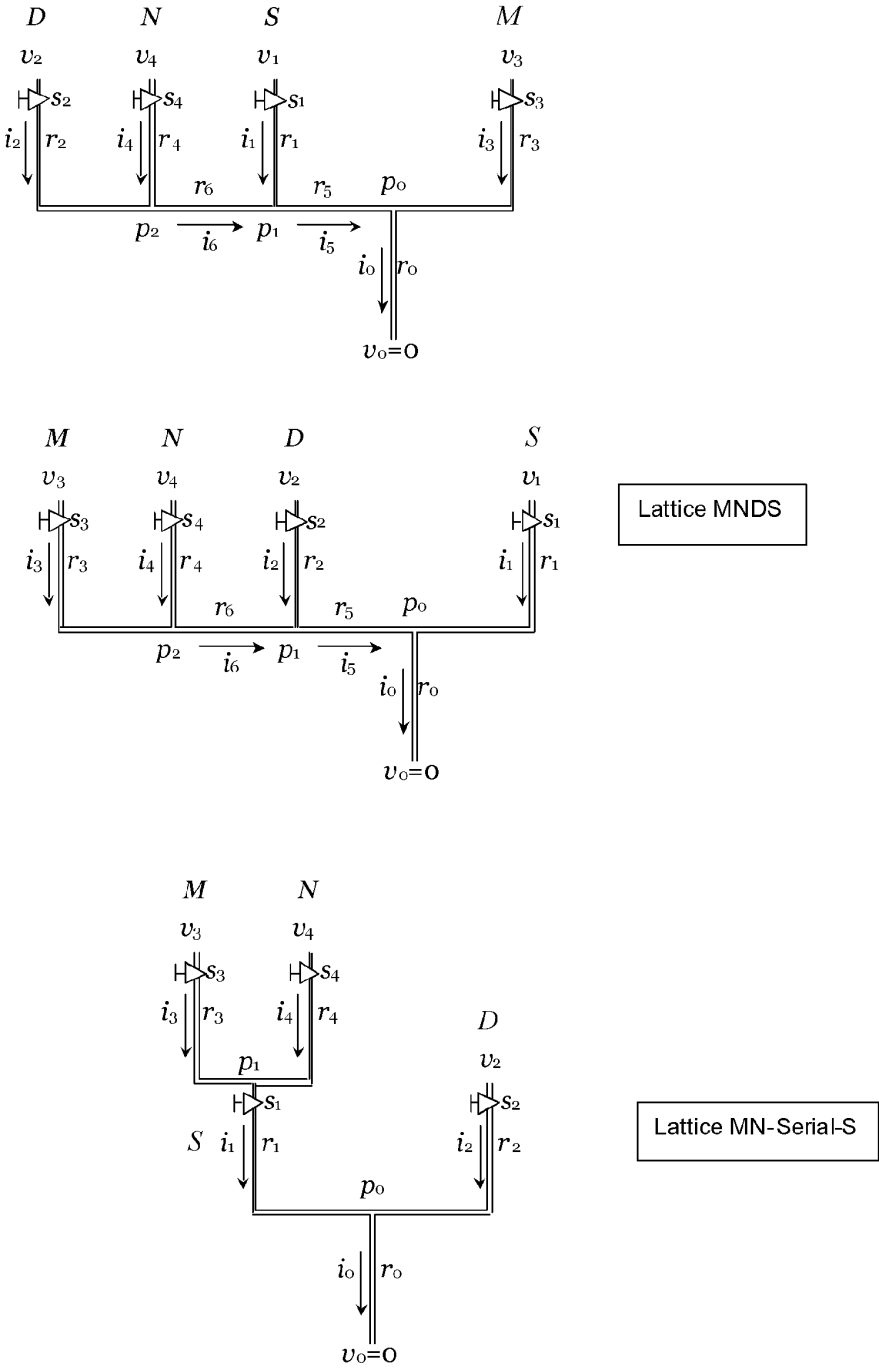
Alternative lattice arrangements. Species “D” and “M” in the upper panel and species “D” and “S” in the middle panel have been switched from that shown in Figure 2B, and in the lower panel the parallel-connected “M” and “N” branches are connected serially to the “S” branch.

This optimization procedure permits multiple optimal states and does not guarantee a global minimum in a reasonable amount of computational time. Hence, the results of this procedure, i.e. the determined model parameters, are understood to be neither unique nor physical, measurable quantities. Rather, the “optimal” set comprises one possible fully specified lattice (connection scheme and model parameters) that is capable of replicating a corresponding set of experiments to within the observed discrepancy. Different choices of the model parameters might produce equally good, or even better, agreement between model and experiment depending on the computational effort expended in their computation. Thus, the procedure is intended to provide a means of assessing the plausibility of a model by bringing to light internal inconsistencies or conflicts. In such event, the user could then alter the lattice connections and rerun the algorithm for all clusters to assess whether alternative lattice arrangements provide a better fit to the experimental data (Figure 3).

The four-branch pipe-lattice model representing TFIID, SAGA, Mot1, and NC2 (Figure 2B) comprises 10 equations for each valve-setting state. Three equations define the flow continuity conditions at the pipe intersections where the pressure head is denoted *p_i_*, *i* = 0,1,2:
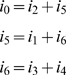
(13)Two equations define the pressure-head drop relations across the pipes whose resistances are denoted *r*_5_ and *r*_6_:
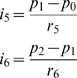
(14)Five equations define the pressure-head drop relations across the pipes whose resistances are denoted *r_i_*, *i* = 0,…,4:
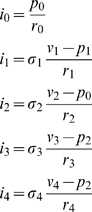
(15)Here too *i*_0_(*σ*_1_, *σ*_2_, *σ*_3_, *σ*_4_) denotes the difference of the mRNA outflow for the valve setting (*σ*_1_, *σ*_2_, *σ*_3_, *σ*_4_) and the mRNA outflow with all valves closed.

### Optimal Solution of the Four-Branch Model for the Model Parameters

Like the two-branch model, the model parameter *r*_0_ defines a class of solutions realized by scaling the values of *v_i_*, *q_i_*, and *r_i_* with *r*_0_; hence, without any loss of generality, we arbitrarily set the value of *r*_0_ to 1. Using any real, positive value of *r*_0_ will produce effectively the same flow in the lattice by the corresponding scaling of the model parameters and internal pressure drops. This model permits a total of 2^4^ = 16 states corresponding to the combination of on/off settings of the four valves *s_i_*, *i* = 1,…,4. However, in contrast to the two-branch model, here the model is overdetermined in the sense that there are 15 non-background experimental values of *i*_0_ (the difference of mRNA outflow for a given state minus mRNA for the background state) that must be replicated by the model via adjustment of only ten model parameters *v_i_*, *i* = 1,…,4, and *r_i_*, *i* = 1,…,6.

Equations 13–15 permit an analytical solution for *i*_0_ in terms of the model parameters and the valve settings. The resulting expression is supplied to the optimization package, GAMS, requiring that the optimal set of model parameters *v_i_*, *i* = 1,…,4, and *r_i_*, *i* = 1,…6, satisfy the following conditions: 1) The difference between *i*_0_ and the experimentally measured mRNA in excess of its background value for the corresponding valves-setting is minimized in the *L*_∞_ norm (the maximum absolute value over all experiments in a cluster); 2) All model resistances are non-negative: *r_i_*≥0, *i* = 1,…6; 3) The sense of change (increase/decrease) relative to the all-valves-on state (Experiment 15) is preserved.

In the pipe-lattice model, the default (wild type) setting for each valve is either on or off, reflecting whether the modelled biological interaction represents a facilitating or inhibiting interaction, respectively. As such, the lowest measured mRNA output in each cluster is assigned to the “all off” state (Experiment 0 in Table 4), regardless of the mutant status and is representative of the background mRNA level. If multiple states possess values that are experimentally indistinguishable from this smallest value, e.g. Cluster 8 discussed below, we arbitrarily select one of them to correspond to the background state. The effect of turning on one or more valves is computed as the difference of the resulting mRNA outflow from this background value. The mutant status for the “all-off” experiment is then directly linked to these valve settings. For example, in cluster 4 the off state of valve *s*_1_ is represented by the SAGA mutant *spt*3Δ (*S*), the off state of *s*_2_ is represented by the TFIID mutant *taf*1(Δ*TAND*) (*D*), the off state of *s*_3_ is represented by wild type *MOT*1, and the off state of *s*_4_ is represented by wild type *BUR*6 (NC2).

**Table 4:** Modeling a four-branch lattice*.

Experiment	0	1	2	3	4	5	6	7	8	9	10	11	12	13	14	15	
*s*_1_ **(S)**	off	on	off	on	off	on	off	on	off	on	off	on	off	on	off	on	
*s*_2_ **(D)**	off	off	on	on	off	off	on	on	off	off	on	on	off	off	on	on	
*s*_3_ **(M)**	off	off	off	off	on	on	on	on	off	off	off	off	on	on	on	on	
*s*_4_ **(N)**	off	off	off	off	off	off	off	off	on	on	on	on	on	on	on	on	Error
**Cluster 3**																	
Mutant		*S*	*D*	*DS*	*N*	*SN*	*DN*	*DSN*	*M*	*SM*	*DM*	*DSM*	*MN*	*SMN*	*DMN*	*DSMN*	
Measured *i_0_*	2.90	3.46	3.16	3.26	4.76	4.55	4.89	4.31	3.36	4.24	3.99	4.19	4.51	4.30	4.61	4.31	
Calculated *i_0_*	2.90	3.49	3.02	3.54	4.60	4.58	4.61	4.59	3.64	3.96	3.71	4.00	4.58	4.57	4.59	4.58	0.086
C∶E–1	0.00	0.01	−0.04	0.09	−0.04	0.01	−0.06	0.06	0.08	−0.07	−0.07	−0.05	0.02	0.06	0.00	0.06	
**Cluster 4**																	
Mutant	*DS*	*D*	*S*		*DSN*	*DN*	*SN*	*N*	*DSM*	*DM*	*SM*	*M*	*DSMN*	*DMN*	*SMN*	*MN*	
Measured *i_0_*	0.65	0.75	0.66	0.80	0.75	0.83	0.76	0.90	0.76	0.80	0.79	0.79	0.73	0.78	0.81	0.91	
Calculated *i_0_*	0.65	0.71	0.70	0.76	0.75	0.81	0.80	0.86	0.72	0.78	0.77	0.83	0.76	0.82	0.81	0.87	0.059
C∶E–1	0.00	−0.06	0.06	−0.05	0.00	−0.03	0.05	−0.04	−0.05	−0.03	−0.02	0.05	0.04	0.05	0.00	−0.05	
**Cluster 5**																	
Mutant	*DS*	*D*	*S*		*DSN*	*DN*	*SN*	*N*	*DSM*	*DM*	*SM*	*M*	*DSMN*	*DMN*	*SMN*	*MN*	
Measured *i_0_*	1.64	2.49	1.77	2.30	2.31	3.64	2.33	3.86	2.21	2.82	2.32	2.58	2.18	3.22	2.34	3.40	
Calculated *i_0_*	1.64	2.58	1.66	2.60	2.61	3.54	2.63	3.56	2.01	2.86	2.02	2.88	2.48	3.33	2.49	3.35	0.135
C∶E–1	0.00	0.04	−0.06	0.13	0.13	−0.03	0.13	−0.08	−0.09	0.01	−0.13	0.12	0.14	0.03	0.07	−0.01	
**Cluster 6**																	
Mutant	*DS*	*D*	*S*		*DSN*	*DN*	*SN*	*N*	*DSM*	*DM*	*SM*	*M*	*DSMN*	*DMN*	*SMN*	*MN*	
Measured *i_0_*	1.36	2.59	1.55	2.60	1.79	2.81	1.75	3.18	1.66	2.40	1.82	2.61	1.62	2.51	1.78	2.97	
Calculated *i_0_*	1.36	2.53	1.56	2.73	1.68	2.84	1.88	3.05	1.53	2.53	1.73	2.73	1.64	2.64	1.84	2.84	0.079
C∶E–1	0.00	−0.02	0.01	0.05	−0.06	0.01	0.08	−0.04	−0.08	0.06	−0.05	0.05	0.01	0.05	0.03	−0.04	
**Cluster 8**																	
Mutant	*DS*	*D*	*S*		*DSN*	*DN*	*SN*	*N*	*DSM*	*DM*	*SM*	*M*	*DSMN*	*DMN*	*SMN*	*MN*	
Measured *i_0_*	2.63	3.50	3.00	3.75	2.77	2.99	2.83	3.18	2.68	2.99	2.86	3.39	2.64	2.88	2.68	3.14	
Calculated *i_0_*	2.63	3.49	2.99	3.85	2.67	3.09	2.84	3.27	2.63	3.09	2.82	3.29	2.66	2.97	2.78	3.09	0.038
C∶E–1	0.00	0.00	0.00	0.03	−0.04	0.04	0.00	0.03	−0.02	0.04	−0.01	−0.03	0.01	0.03	0.04	−0.02	
**Cluster 9**																	
Mutant	*DM*	*DSM*	*M*	*SM*	*DMN*	*DSMN*	*MN*	*SMN*	*D*	*DS*		*S*	*DN*	*DSN*	*N*	*SN*	
Measured *i_0_*	7.21	7.76	8.24	8.37	8.05	8.13	7.99	8.15	8.27	7.45	8.10	8.26	8.54	8.47	8.59	8.84	
Calculated *i_0_*	7.21	7.34	7.87	7.95	8.10	8.10	8.41	8.41	7.85	7.87	8.26	8.27	8.12	8.12	8.42	8.42	0.056
C∶E–1	0.00	−0.05	−0.04	−0.05	0.01	0.00	0.05	0.03	−0.05	0.06	0.02	0.00	−0.05	−0.04	−0.02	−0.05	

* Similar to Table 3, except that the four-branch lattice in Figure 2B was modeled, data sets were from Huisinga et al. [8], and the valve-settings were adjusted such that “all off” (experiment 0) corresponded to the lowest mRNA output for each cluster.

Next, each valve is turned on, one at a time (experiments 1–15), and assigned to the appropriate mutant status. Thus, in cluster 4 experiment 1, *s*_1_ is turned on (wild type SAGA) while all other valves (*s*_2_, *s*_3_, *s*_4_) remain off (mutant TFIID, wild type Mot1, and wild type NC2). When all valves are turned on TFIID and SAGA are in the wild type state and Mot1 (*M*) and NC2 (*N*) are mutant (experiment 15). This process is applied independently to each cluster, then using the GAMS optimization procedure described above, the model parameters shown in Table 5 are computed. The modelled mRNA output (*i*_0_+background mRNA) computed using these parameters is compared to the measured output in Table 4.

**Table 5:** Four-Branch Model parameters*.

Cluster	*v*_1_	*v*_2_	*v*_3_	*v*_4_	*r*_1_	*r*_2_	*r*_3_	*r*_4_	*r*_5_	*r*_6_
**3**	1.65	1.96	8178.61	4471.41	1.81	15.85	30.18	1258.90	0.00	4792.37
**4**	283.88	401.66	368.35	363.25	4724.09	8029.60	1149.90	2657.79	0.00	2531.68
**5**	1.6E+05	208.31	4.0E+04	4.11	1.8E+05	1.0E+04	4.1E+04	5.49	0.00	4.76
**6**	1.2E+07	8.4E+06	1.4E+06	231.91	9.9E+06	4.1E+07	4.3E+06	476.25	193.17	704.11
**8**	1286.68	4.0E+04	0.07	0.00	1486.84	1.1E+05	0.92	1.10	0.00	0.06
**9**	6459.20	1.67	2.6E+06	6544.99	4.1E+04	1.52	2.9E+06	3101.71	7071.16	0.00

* Model parameters were obtained by minimizing the relative error between the measured and model values of mRNA.

With the exception of cluster 5, the error in the modelled output is well below 10%. This value provides a measure of uncertainty with regards to plausibility of the model in Figure 2B to represent the microarray expression data. The higher error associated with cluster 5 indicates that additional regulatory complexity may be associated with this cluster of genes that is not captured by the model. A similar conclusion was drawn regarding the overall validity of the model and the exception of cluster 5 using a different modelling paradigm [9].

Finally, we applied this tool to assess arbitrary alternative arrangements of the lattice model (Figure 3). These alternative arrangements produced larger error when used to model all clusters (Table 6), suggesting that they are poorer models of the underlying transcription mechanism.

**Table 6:** Maximum error associated with the indicated lattice configuration.

Cluster	Lattice*			
	MNSD	DNSM	MNDS	MN-Serial-S
3	0.086	0.098	0.086	0.407
4	0.059	0.067	0.082	0.184
5	0.135	0.146	0.129	0.341
6	0.079	0.086	0.081	0.475
8	0.038	0.043	0.152	0.299
9	0.056	0.078	0.057	0.166
**Total**	**0.453**	**0.518**	**0.587**	**1.872**

* Lattice configuration is designated by the arrangement of pipes from left to right in Figure 2B (MNSD represents the lattice shown in Figure 2B).

## Discussion

The approach described here provides a tool to help interpret large genomic data sets in the context of a model for transcription complex assembly that has ill-defined reaction steps, rate constants, and reactant concentrations. We applied this fluidics model to a large genome-wide microarray expression profile derived through the perturbation of one central aspect of transcription complex assembly (regulation of the TATA binding protein). The approach provided a measure of plausibility of the proposed model by demonstrating that within experimental error the four-branch model adequately represents the data. The results also illustrate the advantages and limitations of our new model in distinguishing good from poor pipe-lattice connection schemes. This modelling tool is not intended to prove that any particular model is correct, nor is it intended to derive a model for assembly. Rather, it provides a computationally expedient means to assess whether a conceptual model of the system that is grounded in conventional wisdom is inherently consistent with, or contradictory to, genomic microarray data.

## Materials and Methods

Microarray expression data were obtained from public sources [8], [9], [26]. Model parameters were determined via GAMS optimization as described in the text.

## References

[1] Novak K, Mandin H, Wilcox E, McLaughlin K (2006). Using a conceptual framework during learning attenuates the loss of expert-type knowledge structure.. BMC Med Educ.

[2] Davidson EH, Rast JP, Oliveri P, Ransick A, Calestani C (2002). A genomic regulatory network for development.. Science.

[3] Sprinzak D, Elowitz MB (2005). Reconstruction of genetic circuits.. Nature.

[4] Crampin EJ, Schnell S, McSharry PE (2004). Mathematical and computational techniques to deduce complex biochemical reaction mechanisms.. Prog Biophys Mol Biol.

[5] Klamt S, Stelling J, Ginkel M, Gilles ED (2003). FluxAnalyzer: exploring structure, pathways, and flux distributions in metabolic networks on interactive flux maps.. Bioinformatics.

[6] Hoops S, Sahle S, Gauges R, Lee C, Pahle J (2006). COPASI–a COmplex PAthway SImulator.. Bioinformatics.

[7] Barshop BA, Wrenn RF, Frieden C (1983). Analysis of numerical methods for computer simulation of kinetic processes: development of KINSIM–a flexible, portable system.. Anal Biochem.

[8] Huisinga KL, Pugh BF (2007). A TATA Binding Protein regulatory network that governs transcription complex assembly.. Genome Biol.

[9] Huisinga KL, Pugh BF (2004). A genome-wide housekeeping role for TFIID and a highly regulated stress-related role for SAGA in Saccharomyces cerevisiae.. Mol Cell.

[10] Millar CB, Xu F, Zhang K, Grunstein M (2006). Acetylation of H2AZ Lys 14 is associated with genome-wide gene activity in yeast.. Genes Dev.

[11] Grant PA, Schieltz D, Pray-Grant MG, Steger DJ, Reese JC (1998). A subset of TAF(II)s are integral components of the SAGA complex required for nucleosome acetylation and transcriptional stimulation.. Cell.

[12] Lee TI, Causton HC, Holstege FC, Shen WC, Hannett N (2000). Redundant roles for the TFIID and SAGA complexes in global transcription.. Nature.

[13] Basehoar AD, Zanton SJ, Pugh BF (2004). Identification and distinct regulation of yeast TATA box-containing genes.. Cell.

[14] Poon D, Bai Y, Campbell AM, Bjorklund S, Kim YJ (1995). Identification and characterization of a TFIID-like multiprotein complex from Saccharomyces cerevisiae.. Proc Natl Acad Sci U S A.

[15] Shen WC, Bhaumik SR, Causton HC, Simon I, Zhu X (2003). Systematic analysis of essential yeast TAFs in genome-wide transcription and preinitiation complex assembly.. Embo J.

[16] Dasgupta A, Juedes SA, Sprouse RO, Auble DT (2005). Mot1-mediated control of transcription complex assembly and activity.. Embo J.

[17] Muldrow TA, Campbell AM, Weil PA, Auble DT (1999). MOT1 can activate basal transcription in vitro by regulating the distribution of TATA binding protein between promoter and nonpromoter sites.. Mol Cell Biol.

[18] Cang Y, Prelich G (2002). Direct stimulation of transcription by negative cofactor 2 (NC2) through TATA-binding protein (TBP).. Proc Natl Acad Sci U S A.

[19] Goppelt A, Meisterernst M (1996). Characterization of the basal inhibitor of class II transcription NC2 from Saccharomyces cerevisiae.. Nucleic Acids Res.

[20] Darst RP, Dasgupta A, Zhu C, Hsu JY, Vroom A (2003). Mot1 Regulates the DNA Binding Activity of Free TATA-binding Protein in an ATP-dependent Manner.. J Biol Chem.

[21] Gumbs OH, Campbell AM, Weil PA (2003). High-affinity DNA binding by a Mot1p-TBP complex: implications for TAF-independent transcription.. Embo J.

[22] Chicca JJ, Auble DT, Pugh BF (1998). Cloning and biochemical characterization of TAF-172, a human homolog of yeast Mot1.. Mol Cell Biol.

[23] Brook A, Kendrick D, Meeraus R, Raman R (2005). GAMS: A Users Guide: GAMS Development Corporation.

[24] Drud A (1985). A GRG code for large sparse dyanmic nonlinear optimization problems.. Mathematical Programming.

[25] Drud A (1992). CONOPT - A Large Scale GRG Code.. ORSA Journal on Computing.

[26] Holstege FC, Jennings EG, Wyrick JJ, Lee TI, Hengartner CJ (1998). Dissecting the regulatory circuitry of a eukaryotic genome.. Cell.

