# Correction: Computational Modelling of Genome-Wide Transcription Assembly Networks Using a Fluidics Analogy

**DOI:** 10.1371/annotation/aadfff4b-6947-475c-8121-225ba113adf8

**Published:** 2008-09-12

**Authors:** Yousry Y. Azmy, Anshuman Gupta, B. Franklin Pugh

The fifth word of the title was misspelled. This also affect the article's citation. The correct title should be: Computational Modelling of Genome-Wide Transcription Assembly Networks Using a Fluidics Analogy. The corrected citation is: Azmy YY, Gupta A, Pugh BF (2008) Computational Modelling of Genome-Wide Transcription Assembly Networks Using a Fluidics Analogy. PLoS ONE 3(8): e3095. doi:10.1371/journal.pone.0003095

